# Neuromodulatory effect of vardenafil on aluminium chloride/d-galactose induced Alzheimer’s disease in rats: emphasis on amyloid-beta, p-tau, PI3K/Akt/p53 pathway, endoplasmic reticulum stress, and cellular senescence

**DOI:** 10.1007/s10787-023-01287-w

**Published:** 2023-07-17

**Authors:** Heba H. Awad, Mahmoud A. Desouky, Alaa Zidan, Mariam Bassem, Amaal Qasem, Mona Farouk, Haidy AlDeab, Miral Fouad, Cherry Hany, Nada Basem, Rita Nader, Ashrakat Alkalleny, Verina Reda, Mina Y. George

**Affiliations:** 1 Department of Pharmacology and Toxicology, Faculty of Pharmacy, October University for Modern Sciences and Arts (MSA University), Cairo, Egypt; 2 Department of Pharmacology and Toxicology, Faculty of Pharmacy, Ain Shams University, Cairo, 11566 Egypt; 3 Drug Design Program, Faculty of Pharmacy, Ain Shams University, Cairo, Egypt

**Keywords:** Alzheimer’s, Vardenafil, Senescence, Proteasome activation, PI3K/Akt/p53, Endoplasmic reticulum stress

## Abstract

**Abstract:**

Dysregulation of protein homeostasis, proteostasis, is a distinctive hallmark of many neurodegenerative disorders and aging. Deleteriously, the accumulation of aberrant proteins in Alzheimer’s disease (AD) is accompanied with a marked collapse in proteostasis network. The current study explored the potential therapeutic effect of vardenafil (VAR), a phosphodiesterase-5 inhibitor, in AlCl_3_/d-galactose (d-gal)-induced AD in rats and its possible underlying mechanisms. The impact of VAR treatment on neurobehavioral function, hippocampal tissue architecture, and the activity of the cholinergic system main enzymes were assessed utilizing VAR at doses of 0.3 mg/kg and 1 mg/kg. Additionally, the expression level of amyloid-beta and phosphorylated tau proteins in the hippocampus were figured out. Accordingly, VAR higher dose was selected to contemplate the possible underlying mechanisms. Intriguingly, VAR elevated the cyclic guanosine monophosphate level in the hippocampus and averted the repressed proteasome activity by AlCl_3_/d-gal; hence, VAR might alleviate the burden of toxic protein aggregates in AD. In addition, a substantial reduction in the activating transcription factor 6-mediated endoplasmic reticulum stress was demonstrated with VAR treatment. Notably, VAR counteracted the AlCl_3_/d-gal-induced depletion of nuclear factor erythroid 2-related factor 2 level. Moreover, the anti-senescence activity of VAR was demonstrated via its ability to restore the balance of the redox circuit. The modulation of phosphatidylinositol-3-kinase/protein kinase B/p53 pathway and the reduction of nuclear factor kappa B level, the key regulator of senescence-associated secretory phenotype mediators release, with VAR treatment were also elucidated. Altogether, these findings insinuate the possible therapeutic benefits of VAR in AD management.

**Graphic abstract:**

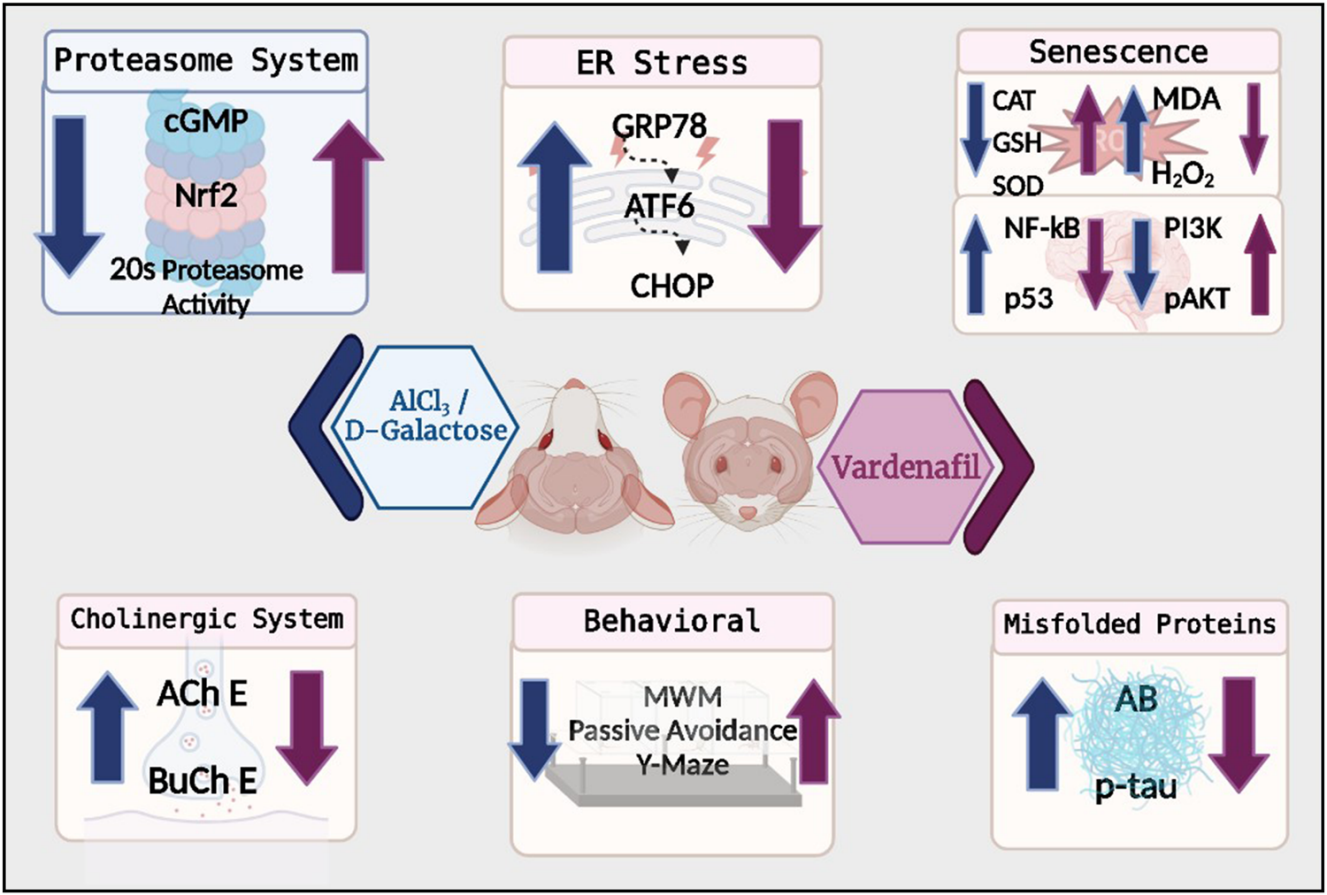

**Supplementary Information:**

The online version contains supplementary material available at 10.1007/s10787-023-01287-w.

## Introduction

Protein homeostasis, proteostasis, is a pivotal process to maintain the proper balance between newly synthesized proteins and eradication of the misfolded protein species; hence, preserving the stability of the entire proteome (Hipp et al. 2019). Importantly, protein misfolding might occur in 30% of the newly generated proteins and the situation is complicated in neuronal cells, being nondividing cells (Höhn et al. 2020). The two main systems that are implicated in sustaining proteostasis and degradation of misfolded proteins are ubiquitin–proteasome system (UPS) and autophagy (Li et al. 2022). Moreover, the dysregulation of proteostasis network might contribute to proteinopathy (Golde et al. 2013). Several studies indicated that the functional capacity of the proteostasis network is deleteriously affected in many neurodegenerative disorders, such as Alzheimer’s disease (AD) in conjunction with aging (Yerbury et al. 2016; Höhn et al. 2020).

AD is a debilitating neurodegenerative disorder characterized by cognitive impairment, as well as marked behavioral alternations, such as depression, apathy, and aggressiveness. Moreover, the aggregation of amyloid-beta (Aβ) and neurofibrillary tangles (NETs) depicts the main pathological features of AD (Silva et al. 2019). Recently, AlCl_3_/d-galactose (d-gal) combination has been utilized to induce AD in animal models. Interestingly, animals treated with these chemicals have shown a prominent decline in cognitive function, abnormal histological changes in hippocampal tissues, aggregation of AD-associated toxic protein species, and deterioration of the cholinergic system (Zhang et al. 2016b; Haider et al. 2020). Additionally, d-gal has demonstrated a deleterious elevation in several senescence markers inducing senescence in animal models (Hou et al. 2019).

A large body of evidence demonstrated that the catalytic activity of the 26S proteasome, the master of the UPS-related protein degradation, is compromised in AD, resulting in accumulation of misfolded proteins (Thibaudeau et al. 2018; Park et al. 2020). Furthermore, these aggregates might activate endoplasmic reticulum (ER) stressors signals, such as the activating transcription factor 6 (ATF6), generating a cascade of unfolded protein response (UPR). ER stress inducers might also cause a significant elevation in level of C/EBP homologous protein (CHOP), which contributes to cellular demise (Lin et al. 2019; Wang et al. 2020).

Detrimentally, AD is associated with a significant elevation in oxidative stress markers with a notable reduction in nuclear factor erythroid 2-related factor 2 (Nrf2) level (Osama et al. 2020). Conjointly, the phosphatidylinositol 3-kinase (PI3K)/protein kinase B (Akt) cellular pathway was reported to be repressed in senescence models (Zhang et al. 2017). It should be emphasized that significant reduction in p53 level, a key regulator of cellular apoptosis, might be attributed to Akt-related signals (Chibaya et al. 2021). Additionally, a notable elevation in senescence-associated secretory phenotype (SASP), which includes a vast array of proinflammatory cytokines was elucidated in senescence models (Kumari and Jat 2021).

Vardenafil (VAR), a phosphodiesterase (PDE)-5 inhibitor, is employed for the treatment of erectile dysfunction (Balasubbramanian et al. 2021). Interestingly, the neuroprotective effect of VAR was demonstrated in a subarachnoid hemorrhage rat model (Gul et al. 2010). Also, sildenafil, a PDE-5 inhibitor, alleviated hippocampal damage, neuroinflammation, and modulated the level of phosphorylated Akt (pAkt) in rat model of neonatal hypoxia–ischemia (Yazdani et al. 2021). Collectively, this study aimed at exploring the potential therapeutic effect of VAR in AlCl_3_/d-gal-induced AD model via reinstating normal protein dynamics and its possible anti-senescence actions.

## Material and methods

### Animals

Male Spargue dawley rats (180 ± 200 g, 8 weeks old) were purchased from Al Roaa Co. for animal breeding, Cairo, Egypt. Rats were left to acclimatize for 14 days before experimentation in an air-conditioned atmosphere (25 °C) with 12 h alternating light and dark cycles. All efforts have been made to minimize animal suffering. Rats were housed in cages with dimensions 80 × 40 × 20 cm^3^. Animals have free access to food and water ad libitum. Animal food consists of carbohydrates (46.4%), proteins (23.6%), fats (3%), and calcium, phosphorous and fibers (27%).

### Drugs and chemicals

VAR was purchased from Bayer Co., for pharmaceutical industries, Cairo, Egypt. AlCl_3_ was purchased from Alfa Aesar Co. (Kandel, Germany). d-gal, Acetylthiocholine iodide, and 5,5′-Dithiobis [2-nitrobenzoic-acid] (DTNB) were purchased from Merck Sigma–Aldrich (St. Louis, MO, USA). For immunohistochemical detection, anti-Aβ and anti-phosphorylated-tau (p-tau) antibody was purchased from ABclonal Technology, MA, US (Catalog No. # A17911 and AP1234, respectively).

### Experimental design

A preliminary study was carried out in which thirty-six rats were randomly assigned into six groups (six rats each) and treated for 10 weeks as follows:

Group I (negative control group) received the vehicles daily for 10 weeks. Group II received AlCl_3_ (200 mg/kg, oral) and d-gal (60 mg/kg, i.p.) daily for 10 weeks. Group III received AlCl_3_ (200 mg/kg, oral) and d-gal (60 mg/kg, i.p.) daily for 10 weeks followed by donepezil (1 mg/kg, oral) daily for 5 weeks starting from the sixth week. Groups IV and V rats were treated with AlCl_3_ (200 mg/kg, oral) and d-gal (60 mg/kg, i.p.) daily for 10 weeks. Then, VAR (0.3 mg/kg and 1 mg/kg, oral, respectively) was administered daily starting from the sixth week. Group VI received VAR (1 mg/kg, oral) daily for 5 weeks starting from the sixth week. The doses for AlCl_3_, d-gal, donepezil, and VAR were selected in accordance with previous studies **(**Akkerman et al. 2014; Chiroma et al. 2019**)**.

Afterwards, rats were subjected to behavioral testing to assess memory functioning as follows: locomotor activity, Morris water maze, step-through passive avoidance, and *y* maze tests. Subsequently, rats were anesthetized, sacrificed by cervical dislocation, skulls were split on ice, and brain tissues were excised. Samples from all groups were fixed in 10% buffered formalin and embedded in paraffin for histological and immunohistochemical examination. Additionally, hippocampi were dissected out and immediately snap frozen in liquid nitrogen and stored at − 80 °C for further biochemical analysis.

Based on the results obtained, VAR (1 mg/kg) was selected for further mechanistic investigations. Forty rats were randomly distributed among five groups (eight rats each); Groups I, II, and III were treated as mentioned in the preliminary study. Group IV rats received AlCl_3_ (200 mg/kg, oral) and d-gal (60 mg/kg, i.p.) daily for ten consecutive weeks. Then, VAR (1 mg/kg, oral) was administered daily starting from the sixth week. Group V received VAR (1 mg/kg, oral) daily for 5 weeks starting from the sixth week. Then, rats were sacrificed, brain tissues were excised, and hippocampi from all groups were dissected out and homogenized at 1:10 (w:v) in phosphate-buffered saline (pH = 7.4).

### Behavioral assessment

#### Locomotor activity

The locomotor activity was assessed using Opto-Varimex-Mini Model B, Columbus Instruments, Columbus, OH, USA. Rats’ locomotion interrupts IR beams. Locomotor activity was recorded as counts per 5 min (George et al. 2020).

#### Long-term spatial memory

Morris water maze was carried out using the 5-day test model. A platform was placed at the center of a target quadrant in a-four quadrant circular white pool (Neuroscience, Osaka, Japan) where its location was kept in changes during the training sessions. Training sessions were performed for five consecutive days for 90 s per rat. On day 5 after the last training session by 3 h, the platform was removed and each rat was placed in the quadrant opposite to the target quadrant and time spent in the target quadrant was monitored and expressed as probe trial (El-Din et al. 2023).

#### Short term memory

Step-through passive avoidance task was adopted to assess rats’ short term memory using Ugo Basile (Italy) apparatus. It is a-two compartment (dark and light) apparatus separated by automatically sliding door. The dark compartment is supplied by an electric current. In the training session, each rat was gently placed in the light compartment and upon entering the dark compartment, the door was closed, an electric shock (0.5 mA–2 s) was conducted, and the cut-off time 90 s. Twenty-four hours later, the testing session (no electric shock) began where rats’ latency to step-through to the dark compartment was recorded indicating memory acquisition, (cut-off time = 300 s) (Ayoub et al. 2022).

#### Short term spatial memory

To perform this task, a *y*-shaped black wooden maze with three similar arms was used. Each rat was placed at the intersecting point of the y maze and left to acclimatize for 5 min. Then, rats’ behavior and arm entries for each rat were recorded. Results were expressed as total arm entries (TAE) and spontaneous alternation percentage (SAP) where SAP = [(number of alternations)/(TAE − 2)] × 100 (George et al. 2023).

### Histological examination

Using a slide microtome, tissue paraffin blocks were partitioned into 4-μm-thick sections, which were placed on glass slides. After deparaffinization, hematoxylin and eosin staining was conducted and hippocampi from different groups were visualized using Leica Microscope DM500, Wetzlar, Germany (Bancroft and Gamble 2008).

### Acetylcholinesterase (AChE) and butyrylcholinesterase (BuChE) activity determination

AChE activity was detected in hippocampi homogenates (Ellman et al. 1961). Briefly, AChE could hydrolyze acetylthiocholine iodide to yield thiocholine, which gives a yellow color in the presence of DTNB detected at 405 nm. Results were expressed as nM/min/gm tissue. BuChE activity was measured using kit (Catalog No. # CE 1240) purchased from Biodiagnostics, Giza, Egypt. Results were expressed as nM/min/gm tissue.

### Immunohistochemical detection of Aβ and p-tau

Following deparaffinization and blocking of 4-μm thickness tissue sections, incubation overnight (4 °C) with both rabbit monoclonal anti-Aβ and rabbit polyclonal anti-p-tau antibodies was performed. Then, biotinylated secondary antibody and streptavidin-HRP were incubated one after the other. Hematoxylin was used for counter staining and the slides were examined using Leica Microscope DM500, Wetzlar, Germany.

### Assessment of cyclic guanosine monophosphate (cGMP) level

Quantification of cGMP in hippocampi homogenates was performed using commercial ELISA kit purchased from Biovision, Inc., CA, USA, (Catalog No. # E4717-100). The assay was carried out in accordance with the manufacturer’s instructions. Results were expressed as pmol/mg protein.

### Proteasome activity determination

A proteasomal activity fluorimetric kit (Cambridge, UK, catalogue No. # ab107921) was used to assess chymotrypsin-like 20S proteasome activity. The intensity of the fluorescence generated from the cleavage of the fluorogenic peptide substrate (Suc-LLVY-AMC) was detected at excitation/emission = 380/460 nm and used as an indicator for proteasomal activity. Results were expressed as RFU/mg protein.

### Assessment of ER stress markers

Determination of ATF6, glucose-regulated protein 78 (GRP78), and CHOP levels was carried out using ELISA kits obtained from FineTest, China (Catalogue No. # ER2065), Life span Biosciecnes, Inc., WA (catalogue No. # LS-F38152), and Sunlong Biotech Co, China (Catalogue No. # SL1663Ra), respectively. All procedures were conducted according to the manufacturer’s protocol. Results were expressed as pg/mg protein for ATF6 and CHOP and as ng/mg protein for GRP78.

### Assessment of Nrf2 level

Nrf2 was determined using ELISA kit purchased from FineTest, China (Catalogue No. # ER962), respectively. All procedures were performed in line with the manufacturer’s procedures and results were expressed as pg/mg protein.

### Senescence markers determination

#### Assessment of PI3K, pAkt, and p53 levels

According to the manufacturer’s protocol, the hippocampal levels of PI3K, pAkt, and p53 were estimated using commercial ELISA kits purchased from FineTest, China (Catalog No. # ER1837), MyBioSource, San Diego, USA (Catalog No. # MBS7254603), and Cusabio, USA (Catalog No. # CSB-E08336r), respectively.

#### Assessment of oxidative stress markers

Using kits obtained from kits from Biodiagnostics, Giza, Egypt, redox markers were detected colorimetrically. The assessment of the antioxidant catalase (CAT) enzyme in the hippocampi of different groups was in accordance with Aebi’s method (Aebi 1984). The expression of the enzyme was estimated as unit/mg protein. Moreover, the determination of the reduced glutathione (GSH) level was in consonance with Beutler et al. (1963) and results were expressed as nmol GSH/mg protein. To evaluate the superoxide dismutase (SOD) enzyme activity, its ability to hinder the reduction of nitroblue tetrazolium dye by phenazine methosulphate was used and the color produced was determined at 560 nm over a 5-min period. This method was in agreement with Nishikimi et al. (1972) and results were expressed as U/min/mg protein. Also, according to Aebi (1984) hydrogen peroxide (H_2_O_2_) was estimated in hippocampal homogenates from various groups and results were expressed as nM/mg protein. Eventually, thiobarbituric acid reactive substances measurement as malondialdehyde (MDA) was employed to evaluate the level of lipid peroxidation in accordance with Kei (1978) and the results were expressed as nmol/mg protein.

#### Assessment of p105 subunit nuclear factor kappa B (NF-κB) level

The level of hippocampal p105 subunit of NF-κB was determined using ELISA kits obtained from Elab science biotechnology Inc., USA (Catalog No. #E-EL-R0673). Results were expressed as pg/mg protein.

### Protein determination

Bradford Protein Assay kit (Catalogue No. #SK3041, Ontario, Canada) was employed to calculate the protein concentration. Briefly, tetradentate copper-protein complexes are generated in an alkaline medium. This complex reduces Folin-Ciocalteu Reagent yielding a water-soluble blue colored product measured at 750 nm.

### Statistical analysis

Data were presented as mean ± SD. The results obtained from passive avoidance test (non-parametric) were analyzed utilizing Kruskal–Wallis test followed by Dunn’ as post hoc test. For the rest of data, multiple comparisons were carried out using one-way ANOVA followed by Tukey as a post-hoc test. In all cases, probability values that were less than 0.05 were considered statistically significant. To sketch the graphs, GraphPad Prism software version 8 (GraphPad Software, Inc., La Jolla, CA, USA) was used.

## Results

### Preliminary study

#### Behavioral assessment

##### Locomotor activity

**Fig. 1:**
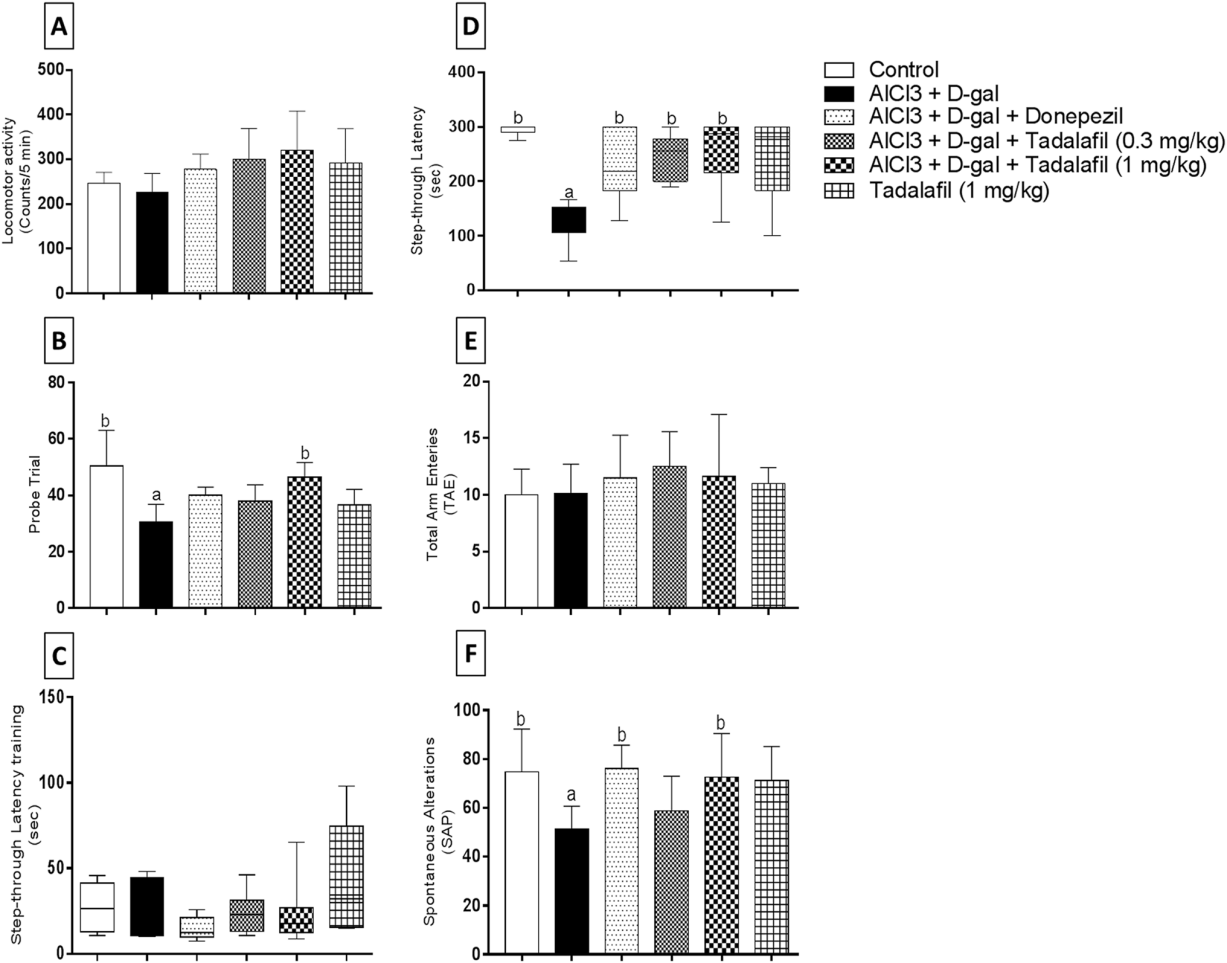
Effect of vardenafil treatment on locomotor activity test (**A**), probe trial testing (**B**), step-through passive avoidance training (**C**), step-through passive avoidance testing (**D**), total arm entries (**E**), and spontaneous alternations (**F**) against AlCl_3_/d-galactose-induced Alzheimer’s disease in rats. Data are presented as mean ± SD (*n* = 6) where: a; statistically significant from control group and b; statistically significant from AlCl_3_/d-galactose-treated group, at *P* < 0.05 using one-way ANOVA followed by Tukey as a post-hoc test (**A**, **B**, **E**, and **F**) and Kruskal–Wallis test followed by Dunn’ post hoc test (**C** and **D**)

One-way ANOVA revealed no significant differences between groups in locomotor activity excluding the effect of locomotion on other behavioral tests (Fig. 1a).

##### Morris water maze

One-way ANOVA showed significant differences between groups in probe trial (Fig. 1b). AlCl_3_/d-gal treatment caused significant reduction in probe trial by 1.65 folds in comparison with the control group. Treatment with VAR (1 mg/kg) revealed a statistically significant increase in probe trial by 1.52 folds relative to AlCl_3_/d-gal-treated group.

##### Step-through passive avoidance

Kruskal–Wallis test revealed no significant differences between groups during the training session (Fig. 1c). Regarding testing session (Fig. 1d), AlCl_3_/d-gal combination was able to reduce step-through latency by 2.31 folds as compared to the control group. Administration of donepezil, VAR (0.3 mg/kg), and VAR (1 mg/kg) significantly enhanced step-through latency by 1.78, 1.92, 1.92 folds, respectively, against AlCl_3_/d-gal-induced group.

##### Y maze

There was no significant difference detected regarding TAE (Fig. 1e). However, AD-induced group presented significant reduction in spontaneous alternations relative to the control group by 1.45 folds. Moreover, treatment with either donepezil or VAR (1 mg/kg) illustrated significant increase in spontaneous alternations by 1.48 and 1.41 folds, respectively, as compared to AlCl_3_/d-gal-treated group (Fig. 1f).

#### Histological examination

**Fig. 2:**
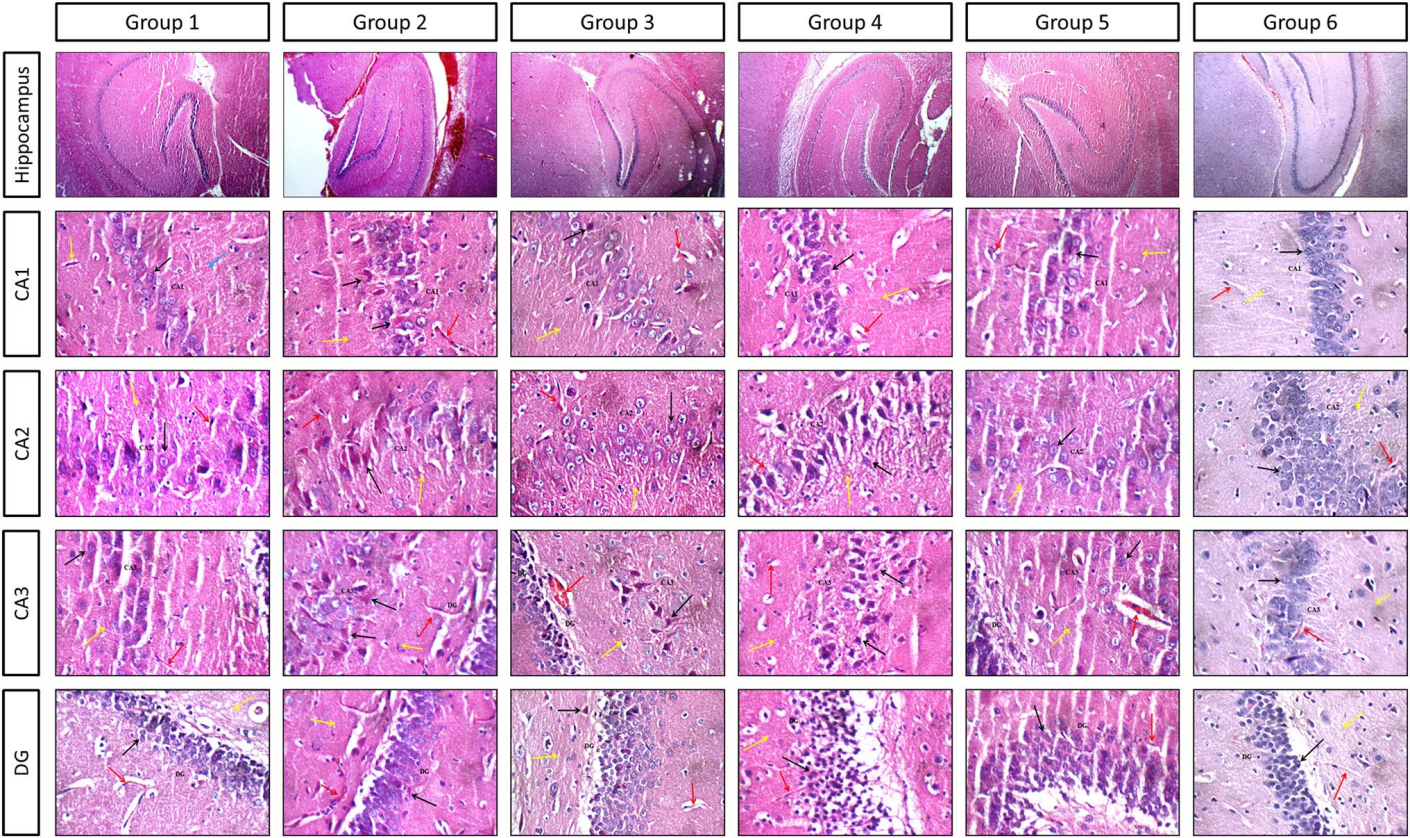
Effects of vardenafil treatment on AlCl_3_/d-galactose-induced histological alterations of rat hippocampal CA1, CA2, CA3, and DG regions. Photomicrographs of haematoxylin and eosin-stained sections from control group (group 1); AlCl_3_/d-galactose-treated group (200 mg/kg, oral) and (60 mg/kg, ip), respectively, (group 2); donepezil-treated group (1 mg/kg) (group 3); vardenafil-treated group (0.3 mg/kg) (group 4); vardenafil-treated group (1 mg/kg) (group 5); vardenafil alone treated group (1 mg/kg) (group 6); with × 100 and × 200 magnification power

Histological examination for hippocampal regions; CA1, CA2, CA3, and dentate gyrus (DG) is presented in Fig. 2. Control group hippocampi showed normal histological structure for the pyramidal neurons, inter-neuron area, and blood vessels for all regions. Group II hippocampi showed scattered degenerated pyramidal neurons in CA1 and DG regions and markedly degenerated pyramidal neurons with eosinophilic plaque-like areas in CA3 and CA2 regions. In addition, mild congested blood vessels were seen in DG region. Treatment with donepezil presented scattered degenerated pyramidal neurons in CA1, CA3, and DG regions, as well as eosinophilic plaque-like areas in CA1 region. Mild congested blood vessels in DG region were also observed. Treatment with low dose VAR (0.3 mg/kg) revealed scattered degenerated pyramidal neurons in CA2 region, markedly degenerated pyramidal neurons in CA3 region, average inter-neuron areas, and average blood vessels congestion. VAR (1 mg/kg) treatment presented scattered degenerated pyramidal neurons and eosinophilic plaque-like area in CA1 region and mildly congested blood vessels in CA3 region. Group VI showed nearly the same records seen in the control group. Histological scoring is recorded in Table 1.

**Table 1:** Histological changes scoring for hippocampus regions among different groups

	Hippocampus					
	Pyramidal neurons				Inter-neuron area	Congested blood vessels
	CA1	CA2	CA3	DG		
Control	0	0	0	0	0	0
AlCl_3_ (200 mg/kg) + d-gal (60 mg/kg)	+	++	++	+	++	+
AlCl_3_ + d-gal + Donepezil (1 mg/kg)	+	0	+	+	++	+
AlCl_3_ + d-gal + VAR (0.3 mg/kg)	0	+	++	0	++	+
AlCl_3_ + d-gal + VAR (1 mg/kg)	+	0	+	0	0	+
VAR (1 mg/kg)	0	0	0	0	0	0

Effect of VAR and donepezil treatment on Alzheimer’s disease induced by AlCl_3_ and d-galactose in rats. AlCl_3_ (200 mg/kg, oral) and d-galactose (60 mg/kg, ip) were administered once daily for ten consecutive weeks. Donepezil (1 mg/kg), VAR (0.3 mg/kg), and VAR (1 mg/kg) were administered once daily for 5 consecutive weeks starting from week six. Scorings of histopathological changes were determined in terms of degenerated pyramidal neurons, inter-neuronal area, and congested blood vessels where

0: indicates normal pyramidal neurons, interneuron area, and blood vessels

+: indicates mild degenerated pyramidal neurons, presence of edema in interneuron area, and mildly congested blood vessels

++: indicates markedly degenerated pyramidal neurons, eosinophilic plaque-like area in interneuron areas, and markedly congested blood vessels

#### AChE and BuChE activity

**Fig. 3:**
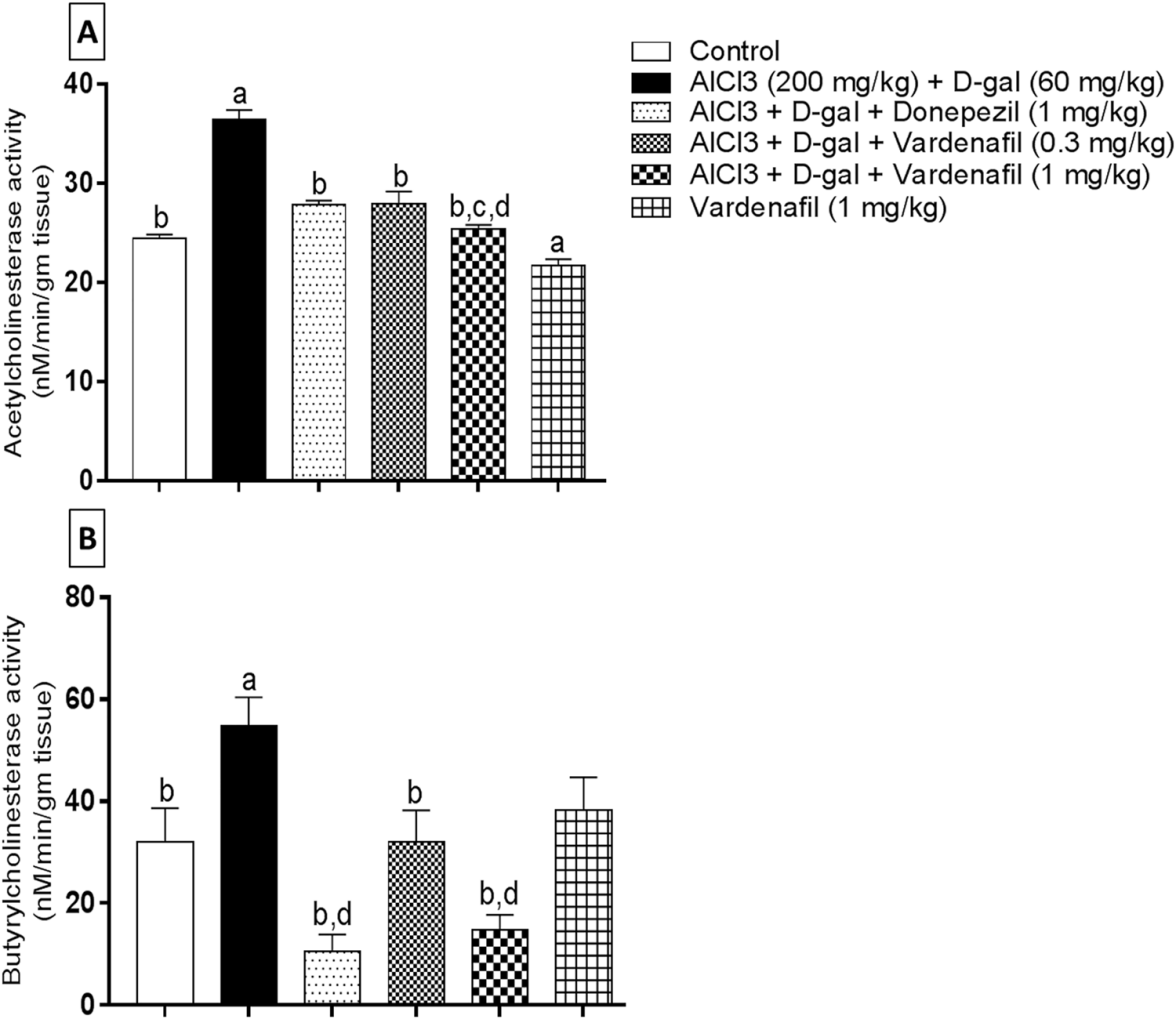
Effect of treatment with vardenafil on hippocampal AChE (**A**) and BuChE activity (**B**) in an experimental model of Alzheimer’s disease induced by AlCl_3_/d-galactose. Data are presented as mean ± SD (*n* = 6) where: a, b, c, and d; statistically significant from control group, AlCl_3_/d-galactose-treated group, donepezil-treated group, and vardenafil (0.3 mg/kg)-treated group, respectively, at *P* < 0.05 using one-way analysis of variance (ANOVA) followed by Tukey as a post-hoc test

Both enzymes activities were estimated in the hippocampi of different groups. As indicated in Fig. 3, AlCl_3_/d-gal caused a significant elevation in both AChE (Fig. 3a) and BuChE (Fig. 3b) activities compared to control group by 1.49 and 1.71 folds, respectively. On the other hand, donepezil, VAR (0.3 mg/kg), and VAR (1 mg/kg) treatment promoted the reduction of both enzymes relative to the disease induced group by 1.31, 1.3, and 1.44 folds, respectively, for AChE and by 5.19, 1.71, and 3.73 folds, respectively, for BuChE. Moreover, VAR (1 mg/kg)-treated rats showed significant reduction in AChE activity relative to both donepezil-treated and VAR (0.3 mg/kg)-treated rats by 1.14 and 1.15 folds, respectively. VAR alone-treated group revealed significant reduction in AChE activity compared to the control group by 1.13 folds. Furthermore, donepezil and VAR (1 mg/kg)-treated groups demonstrated significant reduction in BuChE relative to VAR (0.3 mg/kg)-treated group by 3.04 and 2.19 folds, respectively.

#### Aβ and p-tau expression

**Fig. 4:**
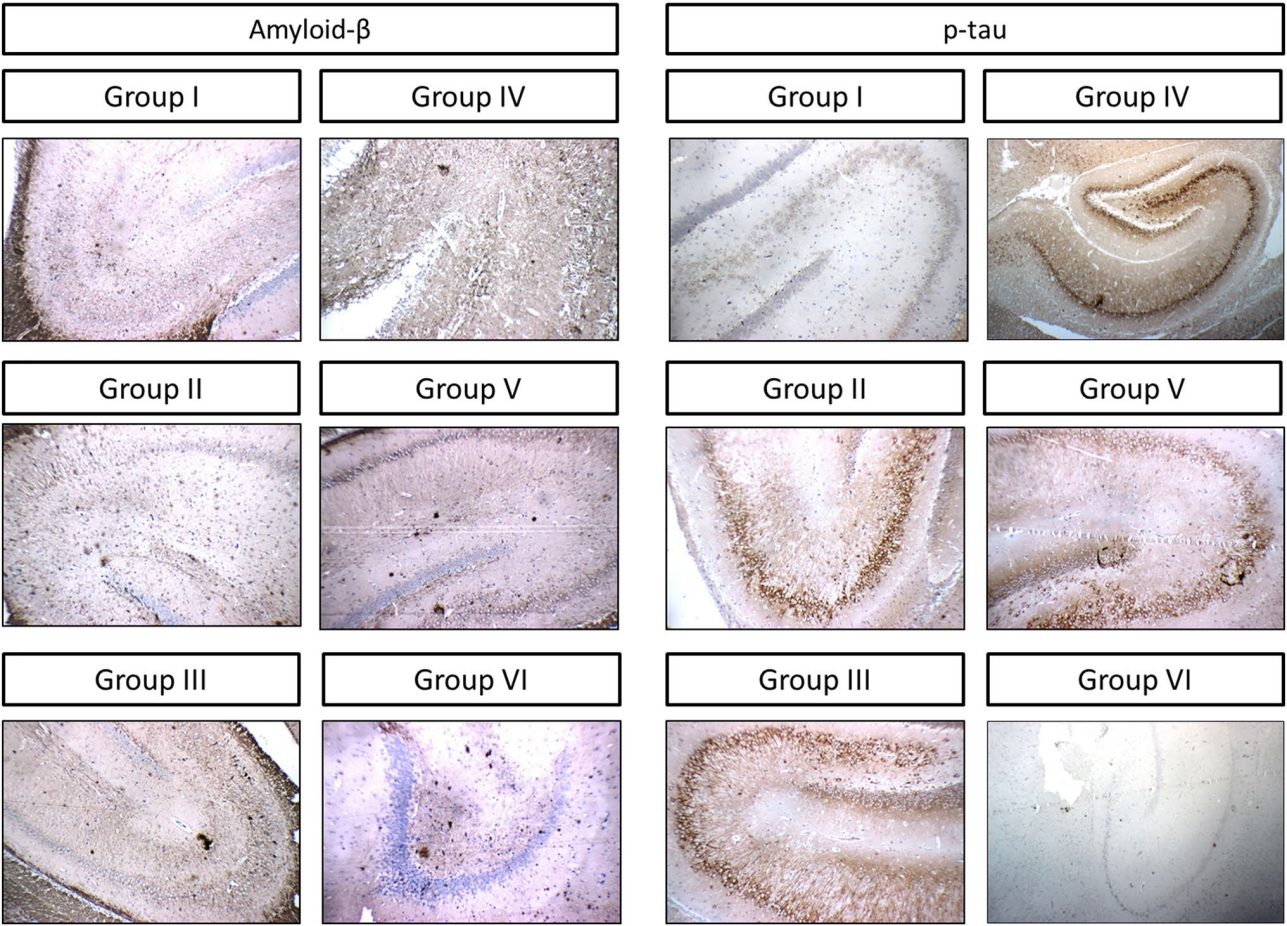
Expression of the hippocampus amyloid-β and p-tau by immunohistochemical staining (× 100). **A** Photomicrographs of histological sections for control group (group I), **B** AlCl_3_/d-galactose-treated group (200 mg/kg) and (60 mg/kg), respectively (group II), **C** donepezil-treated group (1 mg/kg) (group III), **D** vardenafil-treated group (0.3 mg/kg) (group IV), **E** vardenafil-treated group (1 mg/kg) (group V), and **F** vardenafil alone-treated group (1 mg/kg) (group V). Brown color (positive) indicates specific immunostaining of amyloid-β/p-tau and blue color (negative) indicates hematoxyline staining (*n* = 3)

Stained Aβ plaques and p-tau NETs (Fig. 4) were counted in the hippocampi of different rats and recorded in Table 2. Regarding Aβ plaques, significant difference was found between normal control group and group II. Additionally, treatment with donepezil, lower VAR dose, and higher VAR dose significantly lowered the number of accumulated plaques relative to the corresponding AlCl_3_/d-gal-treated group. Interestingly, VAR (1 mg/kg)-treated rats revealed significant reduction in amyloid plaques accumulation relative to the group of rats treated with VAR (0.3 mg/kg) (Suppl. Fig. S1).

**Table 2:** Effect of VAR on amyloid-β plaques and p-tau neurofibrillary tangles in AlCl_3_/d-gal model of AD in rats

Group	Amyloid-β				p-tau				
	CA1	CA2	CA3	DG	CA1		CA2	CA3	DG
Control	–				–				
AlCl_3_ (200 mg/kg) + d-gal (60 mg/kg)	20.17 ± 4.12^a^				45.5 ± 6.72^a^				
AlCl_3_ + d-gal + Donepezil (1 mg/kg)	10.5 ± 3.08^b^				26.5 ± 3.01^b,d^				
AlCl_3_ + d-gal + VAR (0.3 mg/kg)	14.33 ± 2.73^b^				32.17 ± 3.76				
AlCl_3_ + d-gal + VAR (1 mg/kg)	8.01 ± 2.76^b,d^				20.67 ± 3.67^b,d^				
VAR (1 mg/kg)	–				–				

Effect of VAR (0.3 and 1 mg/kg) treatment on amyloid plaques and p-tau tangles in experimentally induced AD in rats. Data are expressed as mean ± SD (*n* = 6). AlCl_3_ (200 mg/kg, oral) and d-galactose (60 mg/kg, ip) were administered once daily for ten consecutive weeks. Donepezil (1 mg/kg), VAR (0.3 mg/kg), and VAR (1 mg/kg) were administered once daily for 5 consecutive weeks starting from week six. a, b, and d: significantly different from control group, AlCl_3_/d-gal-treated group, and AlCl_3_/d-gal + VAR (0.3 mg/kg)-treated group, respectively, at *P* < 0.05 using one-way ANOVA followed by Tukey as a post-hoc test

Concerning p-tau accumulation, group II revealed significant increase in the number of tangles relative to the corresponding control group. However, significant reduction was detected when comparing donepezil and VAR (1 mg/kg) treated rats with both group II and group IV (Suppl. Fig. S2).

### Mechanistic study

#### cGMP level

**Fig. 5:**
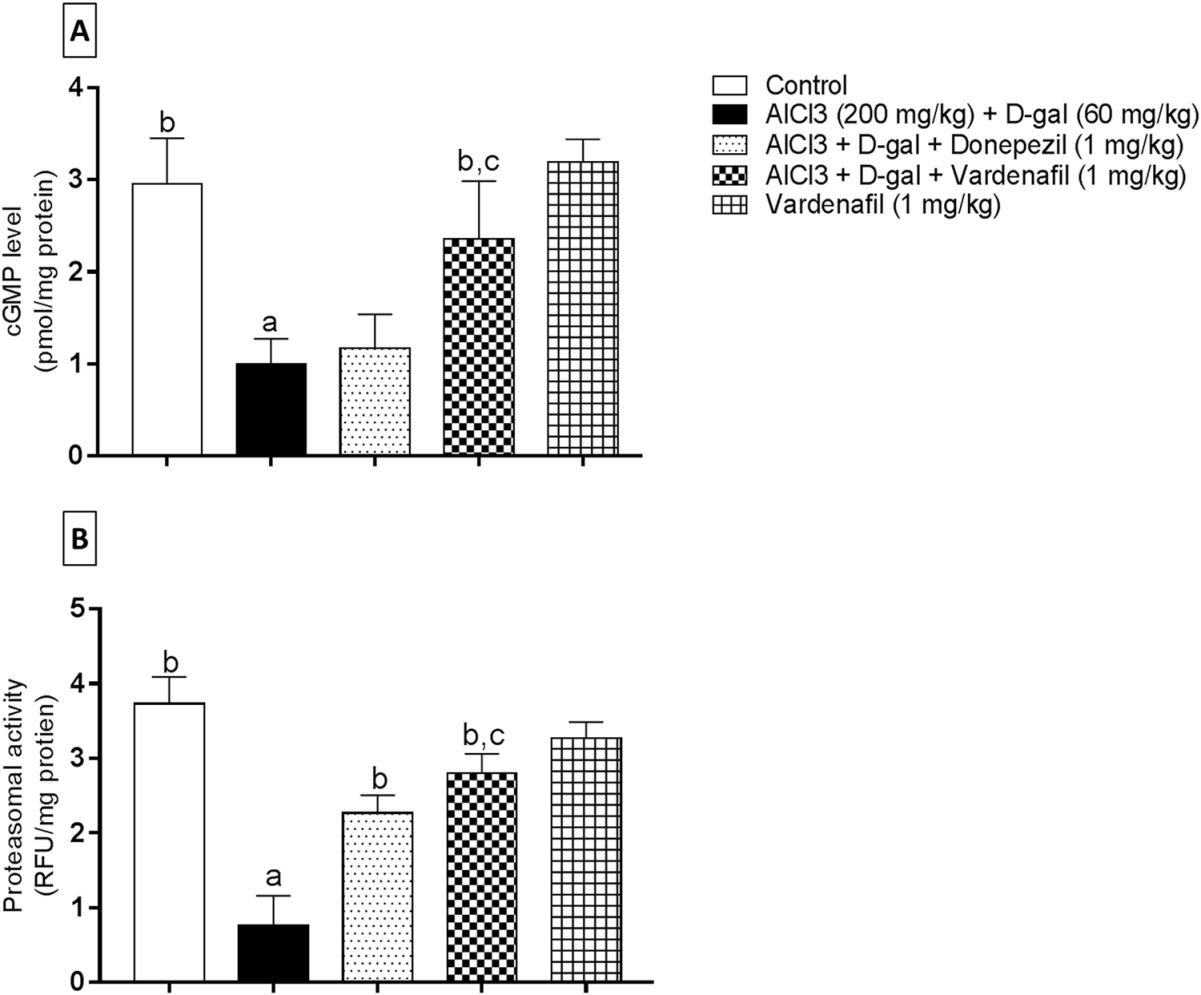
Effect of treatment with vardenafil on hippocampal cGMP level (**A**) and 20S proteasome chymotrypsin-like activity (**B**) in an experimental model of Alzheimer’s disease induced by AlCl_3_/d-galactose. Data are presented as mean ± SD (*n* = 6) where: a, b, and c; statistically significant from control group, AlCl_3_/d-galactose-treated group, and donepezil-treated group, respectively, at *P* < 0.05 using one-way analysis of variance (ANOVA) followed by Tukey as a post-hoc test

The level of cGMP was determined using ELISA technique in hippocampi of different groups to assess its effect on proteasomal activation (Fig. 5a). Data analysis demonstrated that AlCl_3_/d-gal-treated group showed a detrimental suppression in cGMP level by 2.97 compared to the control group. VAR treatment was significantly able to oppose this effect, resulting in a dominant amplification of cGMP levels by 2.36 and 2.01 folds when compared to AlCl_3_/d-gal and donepezil-treated group, respectively.

#### Proteasomal activity

As evident from Fig. 5b, AlCl_3_/d-gal-treated rats exhibited a significant decrease in chymotrypsin-like activity by 4.88 folds relative to the control group. Treatment with either donepezil or VAR was able to reverse such effect in a significant manner compared to the disease-induced group by 2.96 and 3.67 folds, respectively. Interestingly, upon VAR co-administration, the proteasomal activity to degrade the Suc-LLVY-AMC substrate was significantly enhanced by 1.24 folds contrary to donepezil-treated group.

#### ER stress markers

**Fig. 6:**
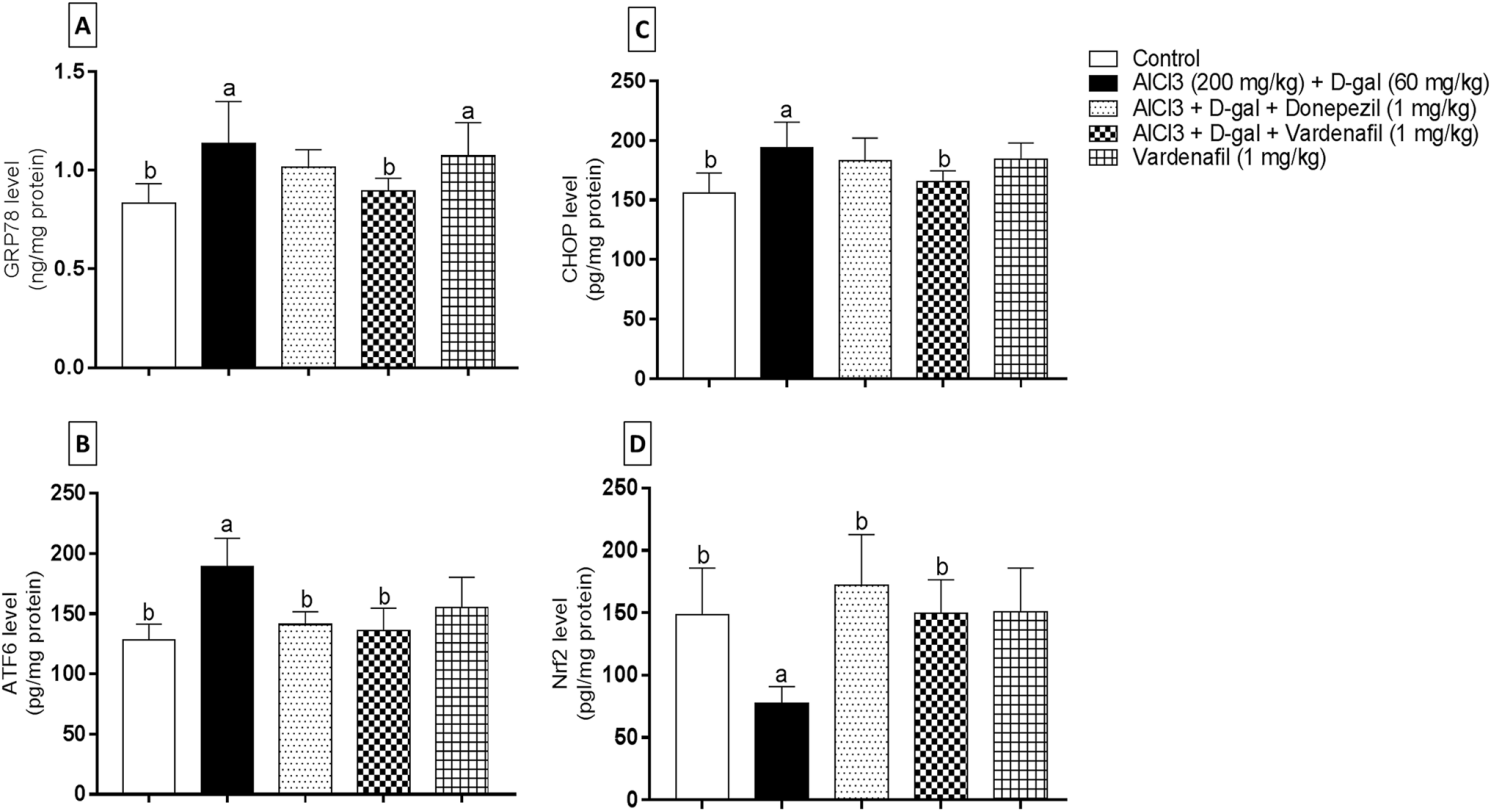
Effect of treatment with vardenafil treatment on hippocampal GRP78 (**A**), ATF6 (**B**), CHOP (**C**), and Nrf2 (**D**) in an experimental model of Alzheimer’s disease induced by AlCl_3_/d-galactose. Data are presented as mean ± SD (*n* = 6) where: a and b; statistically significant from control group and AlCl_3_/d-galactose-treated group, respectively, at *P* < 0.05 using one-way analysis of variance (ANOVA) followed by Tukey as a post-hoc test

As illustrated in Fig. 6a, initial hippocampal GRP78 level was significantly elevated in group II rats by 1.36 folds when compared to the control group. While administration of VAR (1 mg/kg) effectively abrogated GRP78 level by 1.28 folds relative to AlCl_3_/d-gal-treated group. VAR alone-treated rats showed significant elevation in GRP78 level by 1.29 folds relative to the corresponding control group. Afterwards, as shown in Fig. 6b, ATF6 level was found to be significantly elevated in the AlCl_3_/d-gal-treated group by 1.48 folds relative to the control group. On the other side, donepezil and VAR treatment inhibited ATF6 level by 1.34 and 1.39 folds, respectively, in comparison to the disease-induced group. Additionally, one-way ANOVA showed significant augmentation in CHOP level following AlCl_3_/d-gal treatment by 1.24 folds relative to the control group. On the contrary, oral administration of VAR showed marked inhibition in CHOP level by 1.17 folds relative to the group II rats (Fig. 6c).

#### Nrf2 level

The effect of PDE-5 inhibition on Nrf2 level in hippocampi of the different groups was investigated (Fig. 6d). It was found that Nrf2 level in group II was significantly suppressed by 1.92 folds when compared to the control group. Besides, donepezil and VAR (1 mg/kg) treatment significantly enhanced the level Nrf2 by 2.23 and 1.93 folds, respectively, relative to the AlCl_3_/d-gal-treated group.

### Senescence markers

#### PI3K/pAkt/p53 pathway markers

**Fig. 7:**
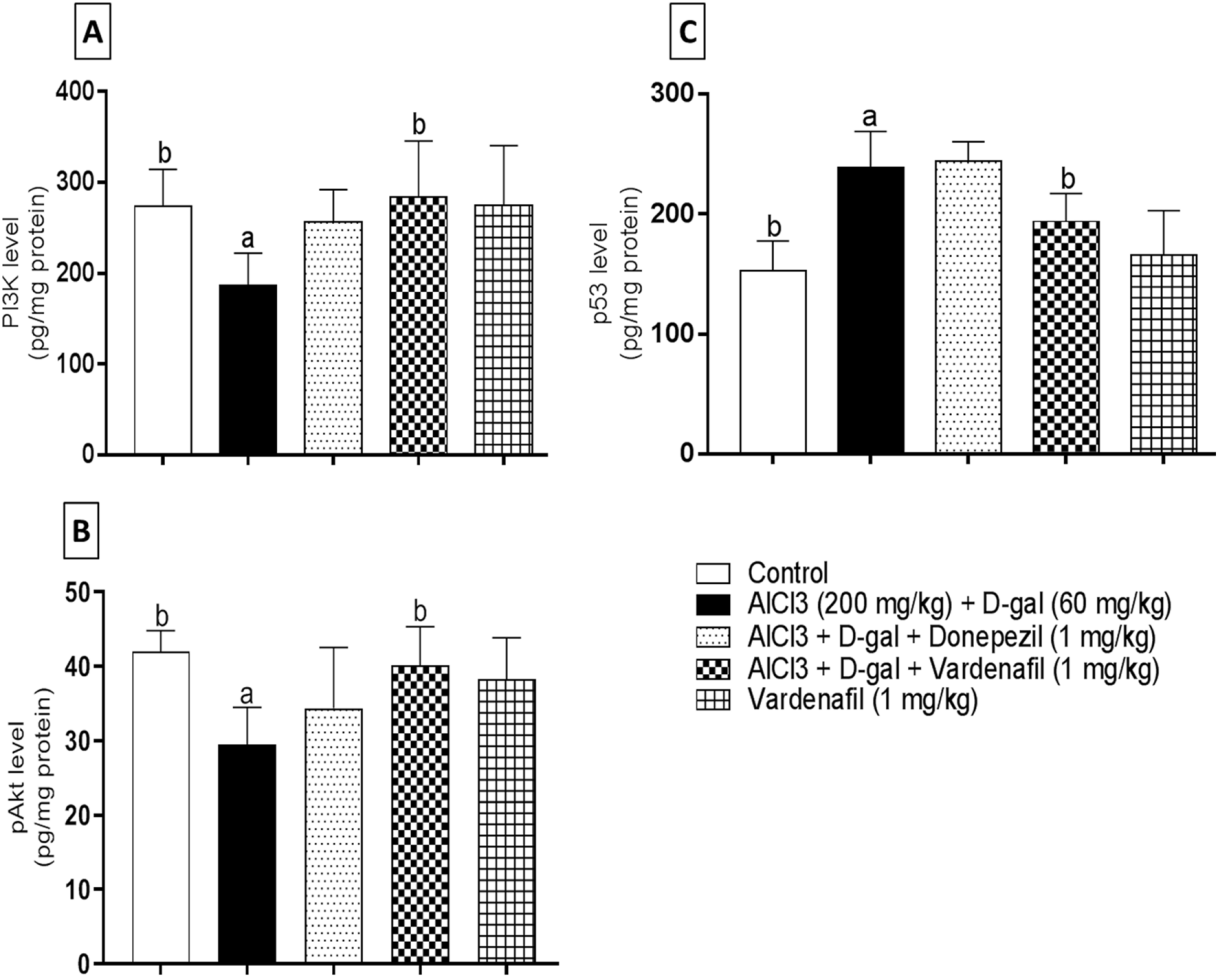
Effect of treatment with vardenafil on hippocampal PI3K (**A**), pAkt (**B**), and p53 (**C**) in an experimental model of Alzheimer’s disease induced by AlCl_3_/d-galactose. Data are presented as mean ± SD (*n* = 6) where: a and b; statistically significant from control group and AlCl_3_/d-galactose-treated group, respectively, at *P* < 0.05 using one-way analysis of variance (ANOVA) followed by Tukey as a post-hoc test

Firstly, hippocampal PI3K level was assessed in different groups (Fig. 7a). Data analysis revealed significant reduction in AlCl_3_/d-gal-treated rats compared to the control group by 1.47 folds. On the other side, VAR treatment showed significant elevation in PI3K level by 1.52 folds relative to AlCl_3_/d-gal-treated group.

Moreover, pAkt level was found to be reduced significantly in hippocampus of group II rats when compared to the control group by 1.42 folds. Moreover, hippocampal pAkt was enhanced in a significant manner following VAR (1 mg/kg) treatment relative to group II rats by 1.36 folds (Fig. 7b).

Finally, the apoptotic p53 level was measured in the hippocampi of different groups (Fig. 7c). A 1.56-fold significant increase was detected in AlCl_3_/d-gal-treated group relative to the control group. By contrast, group of rats treated with VAR showed significant reduction by 1.23 folds compared to disease induced group.

#### Oxidative stress markers

**Fig. 8:**
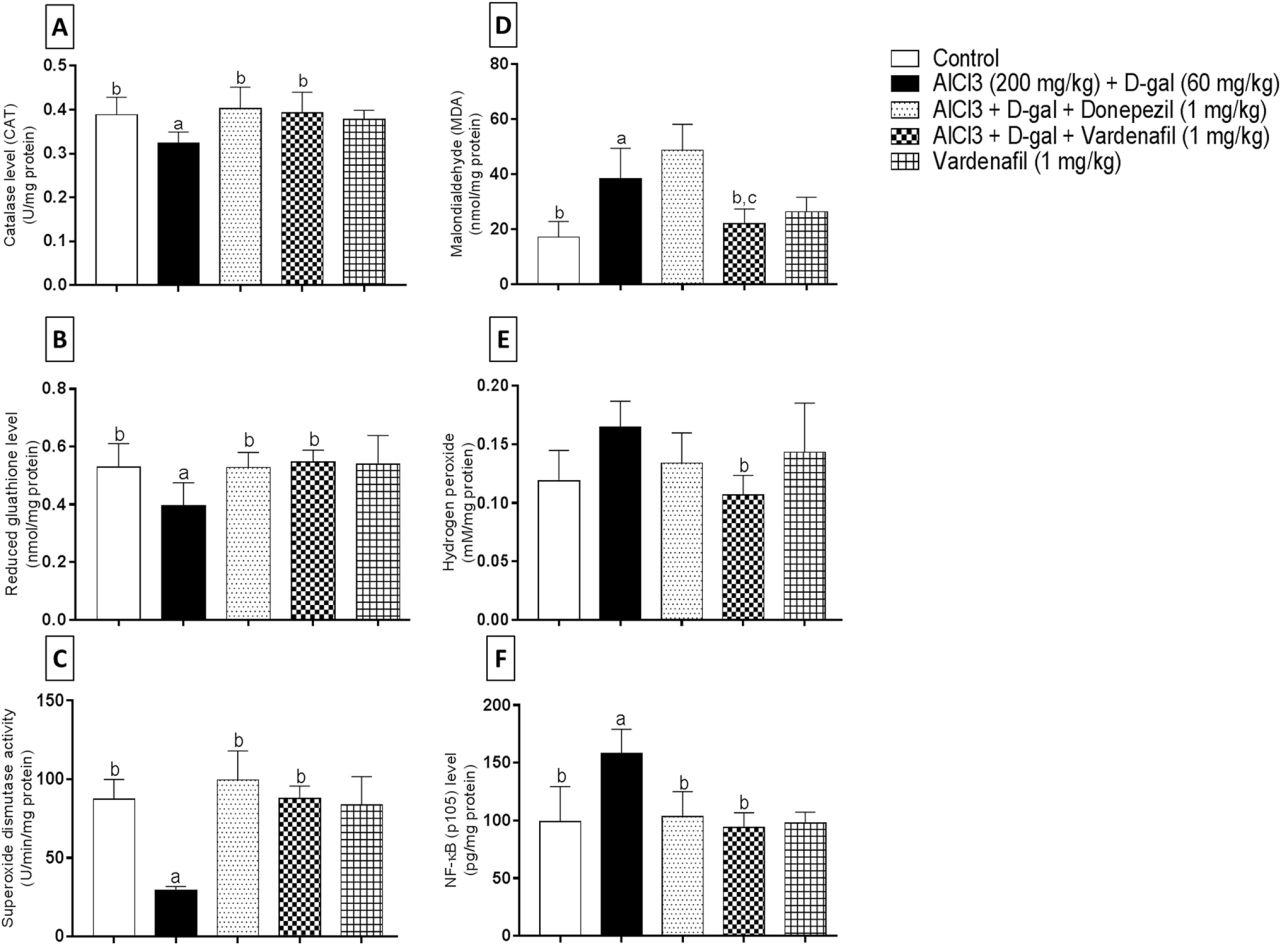
Effect of treatment with vardenafil on hippocampal catalase (**A**), reduced glutathione (**B**), superoxide dismutase activity (**C**), malondialdehyde (**D**), hydrogen peroxide (**E**), and p105 subunit of NF-κB (**F**) in an experimental model of Alzheimer’s disease induced by AlCl_3_/d-galactose. Data are presented as mean ± SD (*n* = 6) where: a, b, and c; statistically significant from control, AlCl_3_/d-galactose-treated group, and donepezil-treated group, respectively, at *P* < 0.05 using one-way analysis of variance (ANOVA) followed by Tukey as a post-hoc test

The levels of CAT, GSH, SOD, MDA, and H_2_O_2_ were assessed in the hippocampi of different treatment groups (Fig. 8). First of all, significant reduction in CAT level was found in AlCl_3_/d-gal-treated group relative to the corresponding control group by 1.21 folds. However, donepezil and VAR treatment opposed this reduction and showed significant elevation in CAT level relative to the disease-induced group by 1.24 and 1.22 folds, respectively (Fig. 8a).

Secondarily, AlCl_3_/d-gal-treated group illustrated a significant reduction by 1.34 folds in GSH level as compared to the control group. Interestingly, treatment with either donepezil or VAR significantly elevated GSH level compared group II rats by 1.33 and 1.38 folds, respectively (Fig. 8b).

Moreover, regarding SOD activity (Fig. 8c), group II rats showed reduced hippocampal SOD activity in a significant manner compared to the control group by 3.01 folds. On the other hand, donepezil and VAR treatment opposed this reduction and enhanced SOD activity significantly relative to AlCl_3_/d-gal-treated rats by 3.42 and 3.02 folds, respectively.

Furthermore, lipid peroxidation level determined as MDA was found to be significantly elevated by 2.24 folds in AlCl_3_/d-gal-treated rats compared to the control group. Interestingly, VAR administration was found to reduce MDA level compared to both group II and donepezil-treated rats in a significant manner by 1.74 and 2.21 folds, respectively (Fig. 8d).

Finally, H_2_O_2_ level was further assessed and one-way ANOVA results revealed an insignificant elevation in H_2_O_2_ in AlCl_3_/d-gal-intoxicated rats relative to the corresponding control group. However, a significant reduction in H_2_O_2_ level was found following VAR administration relative to group II rats by 1.54 folds (Fig. 8e).

#### p105 subunit NF-κB level

As illustrated in Fig. 8f, AlCl_3_/d-gal-treated group of rats revealed significant increase in p105 subunit of NF-κB relative to the control group by 1.59 folds. By contrast, a significant reduction of 1.53 and 1.69 folds was shown when comparing donepezil and VAR (1 mg/kg) treated rats, respectively, to the AD-induced group of rats.

## Discussion

Proteostasis depicts a pivotal cellular process guarantees that properly folded proteins are produced and misfolded proteins are eliminated (Hipp et al. 2019). Normal eukaryotic cells possess an interconnected network known as protein quality control systems, which thoroughly check cellular proteins and detect any fault in their folding to be directed for protein degradation systems (Chen et al. 2011). Interestingly, this surveillance task is mainly attributed to UPS, which is mainly concerned with the elimination of many dysfunctional short-lived proteins linked to ubiquitin (Ub) molecules, as well as autophagy that breakdowns the long-lived proteins (Kocaturk and Gozuacik 2018). Accordingly, any lapse in this ramified network might compromise its integrity, leading to the accumulation of aggregation-prone proteins (Hipp et al. 2019). It is well-established that the capacity of the UPS system is dampened in multiple neurodegenerative disorders and aging (Zheng et al. 2016; Graham and Liu 2017). Scholars have pointed out that misfolded proteins can detrimentally impair the principal degradation mechanisms of the UPS system, resulting in further aggregation of toxic protein species, which collectively creates a vicious cycle wobbles between the collapse of the UPS and proteinopathy (Farizatto et al. 2017; Thibaudeau et al. 2018). To this end, promoting the activity of the UPS might bring a new hope in many neurodegenerative diseases and proteotoxic disorders (Njomen and Tepe 2019).

Basically, AD is a devastating neurodegenerative disorder, which constitutes 60–80% of all cases of dementia. Accumulation of amyloid plaques and NETs are the main pathological features of AD (Abubakar et al. 2022). The amyloidogenic Aβ, a well-known player in amyloid plaques, is generated from Aβ-precursor protein by β-secretases and γ-secretases enzymes while NET might arise from the hyperphosphorylation of tau protein (Knopman et al. 2021; Abubakar et al. 2022).

A growing body of evidence suggests that elevated brain burden of AlCl_3_ could be associated with excessive free radical production, cognitive impairment, and attenuation in the cholinergic system functionality in the hippocampus; hence, it mimics the pathological features of AD (Singh et al. 2018). Importantly, continuous exposure to AlCl_3_ leads to the appearance of Aβ aggregates and NFT alongside with the development of significant oxidative damage and ER stress (Ahmad Rather et al. 2019; Promyo et al. 2020). Recently, d-gal has been identified as a potential chemical that can be utilized to evoke aging via elevating the advanced glycation end product and reactive oxygen species (ROS) production (Rahman et al. 2022). Furthermore, d-gal hinders neurogenesis in the hippocampus with a marked cognitive impairment; thereby, it represents an effective substrate to study aging in animal models (Nam et al. 2019). Consequently, administration of AlCl_3_/d-gal elicited a notable biochemical and behavioral changes that are similar to human AD pathophysiology and thus constitutes a robust AD model (Wei et al. 2017; Chiroma et al. 2018).

To investigate the potential therapeutic impacts of PDE-5 inhibitor, VAR, in alleviating the burden of toxic agglomerates; a preliminary study was firstly conducted to determine the VAR optimal therapeutic dose in our AD model. Importantly, donepezil, was used in this study as a standard treatment for AD to solidify our hypothesis (Haider et al. 2020). Initially, cognitive deficits and memory impairment were observed with rats intoxicated with AlCl_3_/d-gal, which was consistent with previous studies (Haider et al. 2020; Liu et al. 2022). Curiously, VAR enhanced memory, learning abilities, and cognition. This improvement in the neurobehavioral function with VAR high dose treatment was evidenced by increasing the time spent in the target quadrant in Morris water maze, escalation of step-through latency, and improved spontaneous alternations in y maze test. It is worthy to mention that VAR higher dose showed superior results concerning long-term spatial memory (Morris water maze) and short-term spatial memory (y maze) relative to the lower dose. Relevantly, tadalafil, PDE-5 inhibitor, treatment resulted in a marked improvement in memory in a rat model of hyperhomocysteinemia induced vascular dementia (Bhatia and Singh 2022).

Secondarily, histopathological examination of CA1, CA2, CA3, and DG hippocampal regions was thoroughly performed. Normal histological architecture was observed in control and drug alone groups. Expectedly, exposure to AlCl_3_/d-gal caused a substantial neurodegeneration, loss of neuronal organized arrangement, congested blood vessels, and apoptotic alternations in various hippocampal regions, which was in accordance with previous study utilized this combination to induce AD (Gao et al. 2022). Contradictorily, co-treatment with VAR high dose restored nearly the original neuronal architecture and mitigated the number of degenerated neurons while the VAR low dose mildly alleviated the AlCl_3_/d-gal-induced neurodegeneration. Interestingly, these results were supported by an earlier study proved that cilostazol, PDE-3 inhibitor, abrogated the histological anomalies in AD rat model (Khalifa et al. 2022).

Furthermore, several studies explored the role AChE and BuChE enzymes as critical nodes of the cholinergic system dysfunction in AD. The level of these enzymes is notably increased in AD patients; consequently, agents that can inhibit these enzymes might unveil valuable approach in AD treatment (Mushtaq et al. 2014). Strikingly, co-ingestion of VAR and donepezil counteracted the considerable elevation in the activity of both AChE and BuChE enzymes that was induced by AlCl_3_/d-gal. It is worthy to mention that VAR (1 mg/kg) showed powerful results concerning AChE and BuChE relative to the lower dose (0.3 mg/kg). Interestingly, VAR higher dose administration revealed more AChE inhibition relative to donepezil treatment while they showed nearly same results regarding BuChE activity. The significant reduction in AChE activity was in accordance with Haider et al. (2020).

To consolidate the potential therapeutic effect of VAR high dose, the expression level of the main AD pathological hallmarks was explored. In line with previously documented, the expression level of the toxic protein species, Aβ and p-tau, was markedly boosted with AlCl_3_/d-gal treatment in the hippocampus (Zhang et al. 2016b). However, co-administration of VAR and donepezil abrogated the expression level of Aβ and p-tau NETs, which support the hypothesis that alleviation of misfolded proteins burden might be attributed to the activation of UPS (Goldberg et al. 2021). In our study, both donepezil and VAR high dose demonstrated superior results concerning p-tau deposition than lower VAR dose. Additionally, Aβ deposition was found to be markedly decreased following VAR (1 mg/kg) than VAR (0.3 mg/kg) treatment.

Based on the previous results obtained from the preliminary study regarding; neurobehavioral tests, histological examination, AChE and BuChE activities, Aβ expression, and p-tau accumulation results, VAR high dose was selected to demonstrate the mechanistic study.

In the heart of the UPS system is the 26S proteasome (∼ 2.5 MDa), which represent the executive arm for UPS-mediated misfolded protein degradation (Kaake et al. 2014). Basically, it is a barrel shaped complex that comprises 20S core particle with one or two 19S regulatory particles located at the end of the 20S core. The 20S particle has four heteroheptameric rings assembled together and composed of seven α-subunits, as well as seven β-subunits. Importantly, the β1, β2, and β5 subunits house a substantial caspase-like, trypsin-like, and chymotrypsin-like activity, respectively, that can degrade the Ub-tagged proteins (Kaake et al. 2014; Livneh et al. 2016).

As previously mentioned, the catalytic capacity of the 26S proteasome is surmounted in several proteotoxic disorders and with aging; thus, enhancing the proteasome activity might reveal a bright spot in the management of many neurodegenerative diseases such as AD (Graham and Liu 2017; Thibaudeau et al. 2018). In line with this, this study sheds the light on how proteasome capacity could be activated through the elevation of cGMP level. cGMP is a distinctive secondary messenger that is produced in a variety of cells and tissues. Moreover, cGMP generation is catalyzed via the activation of soluble guanylyl cyclase after its binding with nitric oxide or particulate guanylyl cyclase enzyme as a consequence of its activation with the natriuretic peptides (Friebe et al. 2020). Protein kinase G (PKG) is one of the fundamental effectors of cGMP, which plays a pivotal role in many cellular functions (Francis et al. 2010; Friebe et al. 2020). Consistently, VAR was utilized in this study. Rats intoxicated with AlCl_3_/d-gal showed a marked reduction in cGMP level, which was consistent with a previous study reported that the hippocampal level of cGMP could be reduced in AD with a deleterious alternations in memory (Hesse et al. 2017); however, a clear boost in the cGMP level was demonstrated in the hippocampi of VAR-treated animals. Pertinently, recent studies highlighted the potential impact of vericiguat, soluble guanylyl cyclase activator, in memory acquisition and augmenting hippocampal plasticity in rats (Nelissen et al. 2021).

Furthermore, to consolidate the potential impact of VAR against aggregation-prone proteins in AD, the 20S catalytic activity was evaluated via chymotrypsin-like activity fluorimetric assay. Expectedly, AD-associated misfolded protein aggregates were associated a considerable reduction in the 20S peptidase activity, which comes in agreement with Thibaudeau et al. (2018). Contradictorily, VAR adequately augmented the degradation capacity of the 20S core particle. Also, VAR treatment showed superior results compared to donepezil-treated rats. Such alleviation of the burden of aberrant proteins might be attributed to the upregulated level of cGMP, which activates PKG. Furthermore, cGMP-PKG regulatory circuit activation intensifies the 20S capacity to degrade toxic protein species (VerPlank et al. 2020). Likewise, cAMP-PKA pathway activation was previously reported to broaden the 20S peptidase activity via the phosphorylation of Rpn6 proteasome subunit (Lokireddy et al. 2015). Distinctively, the exact site of phosphorylation of the 26S subunits by PKG protein is still not concluded (VerPlank et al. 2020). Concertedly, these results suggest that restoring the activity of the UPS system and mitigating the level of Aβ and NET misfolded proteins after VAR treatment might delineate unconventional paradigm for AD treatment.

ER is a cardinal cellular organelle implicated in protein quality control via its ability to control precise protein folding and post-translational modifications (Araki and Nagata 2011; Adams et al. 2019a). Importantly, a large body of evidence indicated that accumulation of misfolded protein plaques in many neurodegenerative disorders results in the development of ER stress (Sprenkle et al. 2017; Ghemrawi and Khair 2020). To restore the original protein balance, cells initiate a cascade known as UPR, which is transduced via three transmembrane sensor proteins: double-stranded RNA dependent protein kinase-like ER kinase, inositol-requiring enzyme-1α, and ATF6 (Sprenkle et al. 2017; Adams et al. 2019b).

During normal cellular conditions, these stress detectors anchor themselves to a well-known ER chaperone protein, GRP78, which prevent their activation. However, during stressful conditions, such as misfolded protein burden, they free GRP78 away from the transmembrane sensors allowing UPR signal activation (Zhu and Lee 2015; Bartoszewska and Collawn 2020). ATF6 becomes liberated from the ER to enter the Golgi apparatus. Afterwards, ATF6 is translocated into the nucleus adjusting the expression of many targeted genes involved in protein folding (Chiang et al. 2019). Additionally, sustained ER stress contributes to cellular death (Sano and Reed 2013; Ghemrawi and Khair 2020). CHOP is pro-apoptotic factor with an extremely low level of expression; however, under ER stress conditions the ATF6 arm upregulate CHOP expression (Li et al. 2014; Moriguchi et al. 2019). CHOP itself is tangled with the modulation of expression of several anti-apoptotic and pro-apoptotic genes leading to apoptosis (Li et al. 2014; Hu et al. 2019).

Recently, it is widely accepted that ER stress is a key player in AD as figured out from the postmortem brain tissues of AD patients. Similarly, several ER stress markers were showed to be detrimentally elevated in AD, which can be correlated with Aβ-induced ER stress (Uddin et al. 2020). Additionally, marked alternations in the levels of ER molecular chaperones and folding enzymes are documented with aging (Brown and Naidoo 2012). Likewise, rats received AlCl_3_/d-gal displayed a meaningful ER stress as evidenced by the significant rise in GRP78, ATF6, and CHOP levels as previously reported in rat model of d-gal-induced aging (Dai et al. 2023). VAR properly halted the levels of these ER stress key biomarkers. This VAR-associated ER stress alleviation could be ascribed to the elevation of proteasome activity that led to a notable amelioration of neurotoxic proteins and finally buffering the ER stress. As far as we can tell that this is the first study that revealed novel avenues in treating AlCl_3_/d-gal-induced AD via restoring proteostasis and dampening the ER stress. Moreover, donepezil treatment did not show any significance change concerning the levels of GRP78 and CHOP relative to AlCl_3_/d-gal-treated rats.

Dysfunction of the redox state is one of the classical features of AD and aging (Holubiec et al. 2022; Rahman et al. 2022). It is worth noting that our brain is highly susceptible to the imbalance of this regulatory circuit owing to its attenuated antioxidants safeguards and inherit high rate of oxygen utilization. Deleteriously, mitochondrial damage and elevated level of ROS in AD compromise the capacity of antioxidant enzymes (Cenini et al. 2019). Nrf2 is an essential transcription factor that act as a defensive guard against oxidative stress owing to its ability to regulate the expression of several antioxidant genes (Yang et al. 2021). Primarily, under normal physiological conditions, Nrf2 is arrested into the cytoplasm, ubiquitinated, and degraded via proteasomal-mediated process though during stressful cellular events, Nrf2 enters the nucleus, where it can bind to antioxidant responsive element to trigger many protective pathways (Gugliandolo et al. 2020). Previous studies revealed that Nrf2 is greatly affected in wide array of neurodegenerative disorders, including AD (Cuadrado 2016). A large body of evidence reported that, Nrf2 deficiency might result in a profound increment in senescence markers, neuroinflammation, and oxidative stress in addition to proteinopathy (Cuadrado 2016; Fulop et al. 2018). Interestingly, an earlier study demonstrated that activating Nrf2 might be an effective strategy to attenuate d-gal-prompted neurotoxicity and memory impairment (Sun et al. 2020).

Consonantly, our model showed a notable reduction in hippocampal Nrf2 nuclear fraction after AlCl_3_/d-gal treatment as previously reported (Liu et al. 2022). Alternatively, VAR co-treatment abrogated the reduction of Nrf2 nuclear translocation, which might reveal a striking potential anti-aging activity. Donepezil treatment revealed nearly the same results as VAR. Intriguingly, Nrf2 is implicated in the regulation of expression of several proteasome subunits and a striking enhancement in the proteasome capacity was revealed with Nrf2 enhancers (Kwak et al. 2003; Pajares et al. 2017).

PI3K/Akt signaling circuit, is one of the most pivotal cellular cascades being involved in wide range of neuronal functions, such as survival, neurogenesis, and apoptosis (Long et al. 2021). Recently, a deleterious repression of PI3K/Akt axis in AD was reported. Moreover, restoration of the activity of this pathway, ameliorated Aβ-induced neurotoxicity (Zhang et al. 2016a). Interestingly, AlCl_3_/d-gal treatment depicted a detrimental suppression of PI3k and pAkt levels as previously reported (Li et al. 2016). Consistently, our study revealed that VAR co-treatment abrogated AlCl_3_/d-gal inhibitory effect on PI3K/Akt pathway, which comes in agreement with a previous study denoted that tadalafil might activate PI3K/Akt signaling cascade (Lee et al. 2021). Donepezil treatment showed insignificant change relative to AlCl_3_/d-gal treatment.

Importantly, p53 is one of the well-established senescence-associated markers owning to its ability to orchestrate many downstream targets that regulate cell cycle arrest and apoptosis (Mijit et al. 2020). Our study demonstrated that rats treated with AlCl_3_/d-gal showed a marked elevation in p53 level. Relevantly, upregulation of p53 protein level was highlighted in d-gal-induced senescence model (Sha et al. 2021). VAR treatment halted the p53 elevated level; thus, our study augmented a recent study indicated that Akt might act as a negative regulator of p53 (Chibaya et al. 2021). Donepezil showed no effect on p53 hippocampal level. Additionally, PI3K/Akt signaling cascade might be involved in the detoxification of ROS via regulating the activity of Nrf2 transcription factor (Xu et al. 2022).

The anti-senescence activity of VAR was also emphasized via its ability to buffer oxidative stress in the hippocampus. In the present study, AlCl_3_/d-gal-induced oxidative stress was evidenced by CAT and GSH low levels, as well as low enzymatic activity of SOD; however, lipid peroxidation MDA level was notably elevated in hippocampi of the diseased group. Intriguingly, this deleterious oxidative stress was consistent with previous reports that used AlCl_3_/d-gal combination (Wei et al. 2017; Liu et al. 2022). Notable reduction in oxidative stress was substantiated with VAR co-administration as it promoted the level of CAT and GSH with a marked escalation of SOD enzyme activity. The antioxidant power of VAR was further confirmed via its ability to dampen the levels of MDA and H_2_O_2_, which suggests an additional defensive line against oxidative stress in AD and accelerated aging. It is worthy to mention that VAR treatment revealed significant superior results concerning MDA and H_2_O_2_ compared to donepezil treatment. These results solidified a previous study showed the potential antioxidant activity of VAR (El-Agamy et al. 2018). Curiously, VAR capability to relieve oxidative stress might be a direct consequence of Nrf2 pathway activation and enhanced expression of cytoprotective machineries (Francisqueti-Ferron et al. 2019). Additionally, our results were in consonance with a previous study, which documented that cGMP-PKG circuit might be considered a key activator of Nrf2 (Yu et al. 2018).

A large body of evidence showed that senescence is characterized with a selective cellular phenotype, SASP. Moreover, SASPs classical components encompass wide range of proinflammatory cytokines, matrix metalloproteinases, and various proteases (Kumari and Jat 2021). Mounting evidence has revealed that multiple SASP biomarkers are elevated in AD different models (Dorigatti et al. 2022). One of the key regulators of SASP is NF-κB, which orchestrates gene expression of several pro-inflammatory mediators (Kumari and Jat 2021). Concordantly, our study figured out a substantial elevation in the level of NF-κB p105 subunit in the diseased rats as previously mentioned in Song et al. (Song et al. 2022). Contradictorily, VAR mitigated the level of NF-κB 105 subunit; thus, VAR anti-senescence effect might be a direct cause of its ability to inhibit the release of downstream pro-inflammatory mediators of NF-κB. Donepezil treatment showed nearly similar results as VAR. Additionally, triggering ER stress via ATF6 axis represents a crucial arm in cellular senescence (Kim et al. 2019); thus, ER stress mitigation via VAR treatment might present a possible interpretation for VAR anti-senescence action.

Exposure to AlCl_3_ might induce a deleterious cognitive impairment, hippocampal neurodegeneration, cholinergic dysfunction, and accumulation of amyloid plaques (Chen et al. 2021). Once reached the brain of rats, AlCl_3_ deteriorates axonal transport, long-term potentiation, resulting in profound neurodegeneration (Liaquat et al. 2017). Unfortunately, one of the molecular and pathological limitations of this model is the pathological difference between AD-associated plaques in human and the chemically generated plaques in rats (Rapaka et al. 2022). Also, there was no clinical evidence reported linking AlCl_3_ to the development of AD in humans. Additionally, d-gal administration causes marked pathological changes that mimic the human brain aging process as well as, induction of cholinergic dysfunction; thus, can be used to study the molecular mechanisms of AD (Mahdi et al. 2021). However, d-gal is a sugar and might affect blood glucose level especially with chronic exposure. This may occur via its ability to instigate insulin resistance state (Akhtar et al. 2022; Rapaka et al. 2022). Collectively, d-gal could mimic natural aging process and AlCl_3_ induces neurodegeneration, thus, their co-administration may result in pathological changes that could mimic AD features. Currently, no AD experimental model can entirely mimic human AD pathophysiology; however, knowing the limitations of each model might bring a new hope in battle against this leading neurodegenerative disorder (Blaikie et al. 2022).

## Conclusions

In conclusion, the current study emphasized the potential therapeutic effect of VAR in AlCl_3_/d-gal-induced AD. VAR co-treatment successfully dampened memory deficits and histological anomalies in the hippocampus. Moreover, to the best of the authors’ knowledge, this is the first study, which elucidated the proteasomal modulatory effect of VAR in AlCl_3_/d-gal-induced AD via activation of cGMP-PKG regulatory circuit. Also, significant reduction in ER stress was revealed with VAR treatment. VAR attenuated the Nrf2 depletion and activated the PI3K/Akt pathway. Finally, VAR alleviated oxidative stress and mitigated the key SASP modulator, NF-κB p105 subunit. Hence, VAR repurposing might unveil a previously unseen approach in treating age-related neurological disorders via restoring normal dynamics of various protein quality control systems.

### Supplementary Information

Below is the link to the electronic supplementary material.

**Supplementary Fig. S1**: Expression of the hippocampal regions; CA1, CA2, CA3, and DG, amyloid-β by immunohistochemical staining (× 200). Photomicrographs of histological sections for (Group I) control group, (Group II) AlCl3/d-galactose-treated group (200 mg/kg) and (60 mg/kg), respectively, (Group III) donepezil-treated group (1 mg/kg), (Group IV) vardenafil-treated group (0.3 mg/kg), (Group V) vardenafil-treated group (1 mg/kg), and (Group VI) vardenafil alone treated group (1 mg/kg). Brown color (positive) indicates specific immunostaining of amyloid-β and blue color (negative) indicates hematoxyline staining (*n* = 3). (TIF 16226 KB)

**Supplementary Fig. 2**: Expression of the hippocampal regions; CA1, CA2, CA3, and DG, p-tau by immunohistochemical staining (× 200). Photomicrographs of histological sections for (Group I) control group, (Group II) AlCl3/d-galactose-treated group (200 mg/kg) and (60 mg/kg), respectively, (Group III) donepezil-treated group (1 mg/kg), (Group IV) vardenafil-treated group (0.3 mg/kg), (Group V) vardenafil-treated group (1 mg/kg), and (Group VI) vardenafil alone treated group (1 mg/kg). Brown color (positive) indicates specific immunostaining of p-tau and blue color (negative) indicates hematoxyline staining (*n* = 3). (TIF 15431 KB)

## Data Availability

Data will be available on reasonable request.
