# Supplementary figures and images for: Neuromodulatory effect of vardenafil on aluminium chloride/d-galactose induced Alzheimer’s disease in rats: emphasis on amyloid-beta, p-tau, PI3K/Akt/p53 pathway, endoplasmic reticulum stress, and cellular senescence
Source: Inflammopharmacology. 2023 Jul 17;31(5):2653–73. doi: 10.1007/s10787-023-01287-w (PMC10518298; doi:10.1007/s10787-023-01287-w)

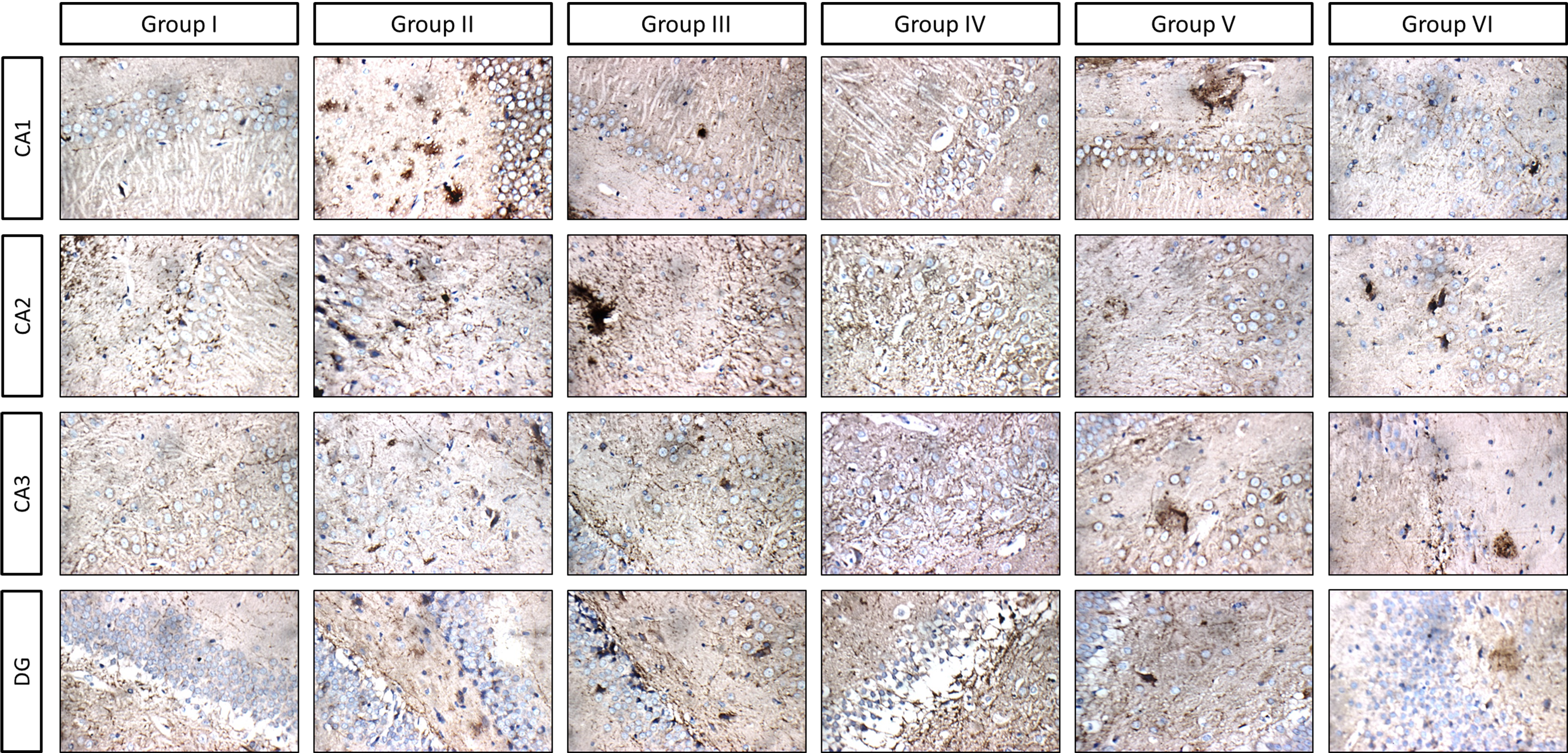

Supplement: Supplementary file 1 — Supplementary Fig. S1: Expression of the hippocampal regions; CA1, CA2, CA3, and DG, amyloid-β by immunohistochemical staining (× 200). Photomicrographs of histological sections for (Group I) control group, (Group II) AlCl3/d-galactose-treated group (200 mg/kg) and (60 mg/kg), respectively, (Group III) donepezil-treated group (1 mg/kg), (Group IV) vardenafil-treated group (0.3 mg/kg), (Group V) vardenafil-treated group (1 mg/kg), and (Group VI) vardenafil alone treated group (1 mg/kg). Brown color (positive) indicates specific immunostaining of amyloid-β and blue color (negative) indicates hematoxyline staining (n = 3). (TIF 16226 KB) [file 10787_2023_1287_MOESM1_ESM.tif]

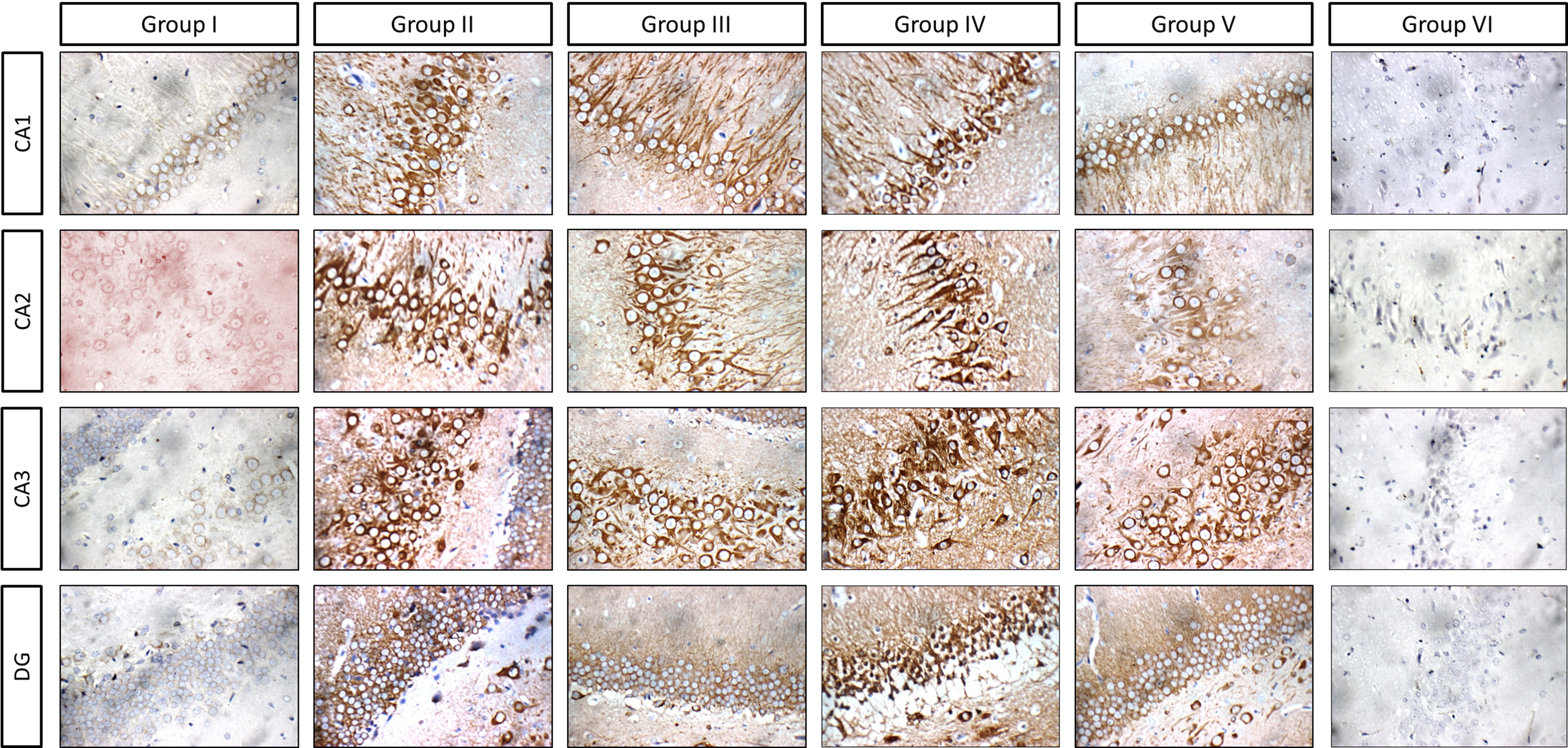

Supplement: Supplementary file 2 — Supplementary Fig. 2: Expression of the hippocampal regions; CA1, CA2, CA3, and DG, p-tau by immunohistochemical staining (× 200). Photomicrographs of histological sections for (Group I) control group, (Group II) AlCl3/d-galactose-treated group (200 mg/kg) and (60 mg/kg), respectively, (Group III) donepezil-treated group (1 mg/kg), (Group IV) vardenafil-treated group (0.3 mg/kg), (Group V) vardenafil-treated group (1 mg/kg), and (Group VI) vardenafil alone treated group (1 mg/kg). Brown color (positive) indicates specific immunostaining of p-tau and blue color (negative) indicates hematoxyline staining (n = 3). (TIF 15431 KB) [file 10787_2023_1287_MOESM2_ESM.tif]
